# Study of the Spatio-Chemical Heterogeneity of Tannin-Furanic Foams: From 1D FTIR Spectroscopy to 3D FTIR Micro-Computed Tomography

**DOI:** 10.3390/ijms222312869

**Published:** 2021-11-28

**Authors:** Nicola Cefarin, Diana E. Bedolla, Artur Surowka, Sandro Donato, Thomas Sepperer, Gianluca Tondi, Diego Dreossi, Nicola Sodini, Giovanni Birarda, Lisa Vaccari

**Affiliations:** 1 Elettra-Sincrotrone Trieste, S.S. 14 Km 163.5, Basovizza, 34149 Trieste, Italy; cefarin@iom.cnr.it (N.C.); diana.bedolla@elettra.eu (D.E.B.); asurowka@agh.edu.pl (A.S.); diego.dreossi@elettra.eu (D.D.); nicola.sodini@elettra.eu (N.S.); lisa.vaccari@elettra.eu (L.V.); 2 IOM-CNR, Trieste, S.S. 14 Km 163.5, Basovizza, 34149 Trieste, Italy; 3 Area Science Park, Padriciano 99, 34149 Trieste, Italy; 4 Faculty of Physics and Applied Computer Science, Department of Medical Physics and Biophysics, AGH University of Science and Technology, al. Mickiewicza 30, 30-059 Kraków, Poland; 5 Department of Physics, University of Calabria, Via P.Bucci 31C, 87036 Rende, Italy; sandro.donato@fis.unical.it; 6 Division of Frascati, Istituto Nazionale di Fisica Nucleare, Via Fermi, 54, 00044 Frascati, Italy; 7 Forest Products Technology & Timber Constructions Department, Salzburg University of Applied Sciences, Marktstrasse 136a, 5431 Kuchl, Austria; thomas.sepperer@fh-salzburg.ac.at (T.S.); gianluca.tondi@unipd.it (G.T.); 8 Salzburg Center for Smart Materials, Jakob-Haringerstrasse 2a, 5020 Salzburg, Austria; 9 Department of Land, Environment, Agriculture & Forestry, University of Padua, Viale dell’Università 16, 35020 Legnaro, Italy

**Keywords:** tannin-furanic rigid foam, FTIR spectroscopy, FTIR imaging, FTIR micro-tomography

## Abstract

Tannin-furanic rigid foams are bio-based copolymers of tannin plant extract and furfuryl alcohol, promising candidates to replace synthetic insulation foams, as for example polyurethanes and phenolics, in eco-sustainable buildings thanks to their functional properties, such as lightness of the material and fire resistance. Despite their relevance as environmental-friendly alternatives to petroleum derivatives, many aspects of the polymerization chemistry still remain unclear. One of the open issues is on the spatial heterogeneity of the foam, i.e., whether the foam constituents prevalently polymerize in spatially segregated blocks or distribute almost homogenously in the foam volume. To address this matter, here we propose a multiscale FTIR study encompassing 1D FTIR spectroscopy, 2D FTIR imaging and 3D FTIR micro-tomography (FTIR-μCT) on tannin-furanic rigid foams obtained by varying the synthesis parameters in a controlled way. Thanks to the implementation of the acquisition and processing pipeline of FTIR-μCT, we were able for the first time to demonstrate that the polymer formulations influence the spatial organization of the foam at the microscale and, at the same time, prove the reliability of FTIR-μCT data by comparing 2D FTIR images and the projection of the 3D chemical images on the same plane.

## 1. Introduction

Fourier Transform InfraRed (FTIR) spectroscopy is a very informative analytical technique that allows to identify chemical moieties of a sample in a non-destructive and label-free way [1,2]. With respect to conventional FTIR spectroscopy, FTIR microscopy exploits the characterization capabilities of FTIR at the microscale, to obtain 2D information on the chemical distribution of the sample [3] with diffraction-limited lateral resolution. In particular, bi-dimensional imaging detectors, such as the Focal Plane Array (FPA), perform well in the Mid-IR regime, granting hyperspectral data on relatively large sample areas and rather short acquisition times. It is therefore not surprising the increased importance gained by FTIR imaging in the last decades, for diverse applications, ranging from infrared spectral histology [4] to polymer [5] and heritage science [6].

Nevertheless, FTIR imaging projects a 3D object onto a 2D plane, limiting diagnostic capabilities of those samples that are not chemically homogeneous along the direction of the optical path. In order to obtain a better representation of the system under analysis, a tridimensional reconstruction might be the solution, achievable by FTIR micro-computed tomography (FTIR-µCT). This relatively new imaging modality, as presented by Martin et al. in 2013 [7], combines FTIR imaging and micro-computed tomography, opening the way for 4D (x, y, z, wavenumber) hyperspectral chemical micro-imaging of complex organic and biomolecular systems. As it happens for X-ray µCT, FTIR-µCT experiments are carried out by rotating the sample under the photon beam and acquiring the respective planar projections. The main difference relies on the presence of an interferometer in the FTIR-µCT setup that allows obtaining at each sample-rotation angle a hyperspectral projection with different chemical contents according to the selected spectral band. Hence, instead of providing upon reconstruction volumes of X-ray attenuation of the object, known as voxels, FTIR-µCT yields chemical volumes, and it is therefore useful to determine the chemical heterogeneity of the sample in accordance with the size of the chemical-voxel. 

To date, just few examples of application of FTIR-µCT have been published, that are mostly proof-of-concept application of the technique to characterize biological samples such as tissues [7] or cells [7,8,9], polymeric blends [10], organic [7] or inorganic materials [11,12]. Indeed, despite being very promising, FTIR-µCT suffers from different constraints, being the first the sample’s suitability for the technique. Since these measurements are typically performed in transmission mode, they require the sample to have an optimal thickness, yielding high signal-to-noise and no saturation in FTIR data, at least for the chemical moieties of interest. Depending on the sample nature, its optimal thickness is typically in the range of few microns. In addition, the sample lateral size in FTIR-µCT experiments is also limited by the field of view, which is dependent on the number of pixels of the detector and the magnification of the objectives used to focus the light onto the specimen. In this case, the size is typically in the range of a few hundreds of microns. Therefore, samples should be pretty small and intrinsically light, in general no “self-supporting”. In the aforementioned published studies with FTIR-µCT, the samples were held within polyimide or nylon micro-loops, which both have a mid-infrared contribution, thus requiring to choose the best option in accordance to the sample’s bands of interest. Another alternative, which may be the best option, is to glue the specimen to a needle-like support [11]. However, it only works for stable and hard materials while it does not for soft biological samples, and in all the cases there is the risk of having the glue interfering with the measurements. 

Highly-porous materials can be indeed investigated with this approach, even when they are relatively thick and, as we have recently proven by presenting the applicability of the technique for the analysis of tannin-furanic rigid foams embedded in paraffin oil [10]. In the aforementioned paper, we also introduced our dedicated acquisition setup, mechanically more stable and precise than most of the ones already reported, and we substantially improved the speed of data acquisition thanks to the dedicated automatic control software. The present work builds up from the past experience and presents progresses that have been made on the optimization of the i- acquisition setup, ii- data acquisition through the minimization of time-loss, iii- quality of both raw and pre-processed data to guarantee the best possible output of the 3D renderings and the fidelity of chemical info therein contained. In continuity with our previous activities, we maintained the focus on the tannin-furanic foams, bio-based, lightweight intelligent materials that aim to replace synthetic insulation foams such as polyurethanes and phenolics in eco-sustainable buildings. They are highly-porous rigid foams made by a network of cells with thin walls and variable size, ranging from few microns to some millimeters, connected through thicker interconnects and knots. As a matter of fact, these completely natural foams combine excellent physical properties such as low thermal conductivity and high fire resistance with relatively low costs [13,14,15,16]. The major constituent of tannin-furanic foams, tannins, are simply extracts of trees or tree bark and are already industrially available because of their extensive use in the tannery raw. The other main component of the backbone of these materials is of furanic nature, derived from furfuryl alcohol, that is also produced from plant carbohydrates [17,18]. Despite their technological relevance and environmental-friendly characteristics, the polymerization pathways that originate the foams are still not fully understood and how they reflect on the spatial heterogeneity of the sample is a topic that has never been addressed yet. In this paper, we considered five different foam formulations, and applied a multiscale approach for their study. Starting from a 1D investigation with Attenuated Total Reflection (ATR) FTIR spectroscopy, and then progressing into a 2D view through FTIR FPA imaging, we finally applied optimized 3D FTIR-µCT protocols to identify if tannin-polyphenols and furanic moieties of the foam are equally distributed all over or if a segregation at the level of the foam volume exists. The proposed multiscale approach is the key-point of the present paper, and it allowed to obtain, for the first time, a 3D chemical representation of tannin-furanic foams and highlight their spatial heterogeneity at the microscale.

## 2. Results and Discussion

**Figure 1:**
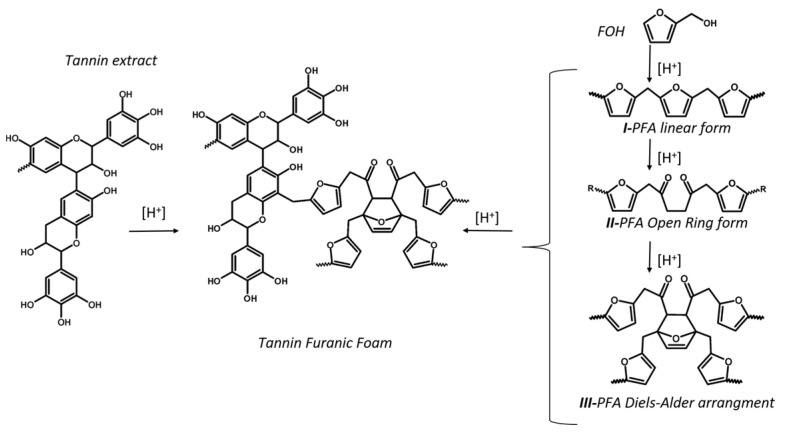
Acid-catalyzed copolymerization of tannin extract and FOH in acid environment to tannin-furanic foam. The most probable homopolymerization pathways of FOH to PFA are reported: I-PFA linear aliphatic structure, II-PFA γ-diketonic Ring-open structure, III-Diels-Alder rearrangement of I and II.

From a chemical point of view tannin-furanic foams are copolymers of two units: the polyphenolics of the tannin plant extract and the furanics derived from the acid-catalyzed polymerization of furfuryl alcohol (FOH). In Figure 1 the possible homopolymerization pathways for FOH are sketched. Specifically, the more probable polyfurfuryl alcohol (PFA) structures attainable in our polymerization conditions, detailed in Section 4.1, are presented (I.-PFA linear aliphatic structure, II.-PFA γ-diketonic Ring-open structure, III.-Diels-Alder rearrangement of I and II) [19,20]. In tannin rigid foam, PFA and tannin-po-lyphenols moieties are covalently bonded [21], but their arrangement can vary from the more homogeneous case, in which tannin-polyphenols and furanics blocks alternate periodically in all parts of the structure (prevalent alternated copolymerization), to the more inhomogeneous one, where polyphenols and furanics blocks are consistently separated in different areas of the structure (prevalent graft copolymerization, where blocks of the homopolymers are grafted). In Figure 1, only one possible copolymer structure derived from III-PFA is presented.

Specifically, if the reaction conditions favor the prevalent homopolymerization of FOH to PFA, extended and almost segregated furanic blocks, rich of furanic rings and carbonyl groups, and extended tannin blocks, rich of phenolics and hydroxy groups, are expected. Conversely, if the alternated copolymerization is favored, an alternation of these blocks is more likely. Of course, all possible shades between these extremes are possible and indeed very probable. The spatial distribution of these polymer blocks in the foam structure will depend not only from its formulation, but also from other reaction parameters such as stirring condition, temperature distribution and reagent’s dispersion. In this study, we focused on the importance of the chemical formulation, by varying the relative proportion of furfuryl alcohol (FOH High and FOH Low formulations) and sulphuric acid (Acid High and Acid Low formulations) with respect to a standard formulation (Reference from here on), as reported in Section 4.1., to observe their impact on the structure of the polymer. It is reasonable to expect that the furanic blocks will be more extended in the furfuryl alcohol-richer foams (FOH High), while, for the acid catalyst-richer foams (Acid High), we expect higher furanic ring-opening, hence Diels-Alder arrangement, but this may involve highly concentrated PFA region with high crosslinking capabilities, carrying to higher chance to copolymerize earlier with the polyphenols bringing to a more homogeneous structure.

In order to verify if the foams are in line with the expectations, we exploited a multiscale approach, based on 1D ATR-FTIR spectroscopy, 2D FTIR imaging and 3D FTIR-µCT.

### 2.1. 1D ATR-FTIR of Tannin Foams

**Figure 2:**
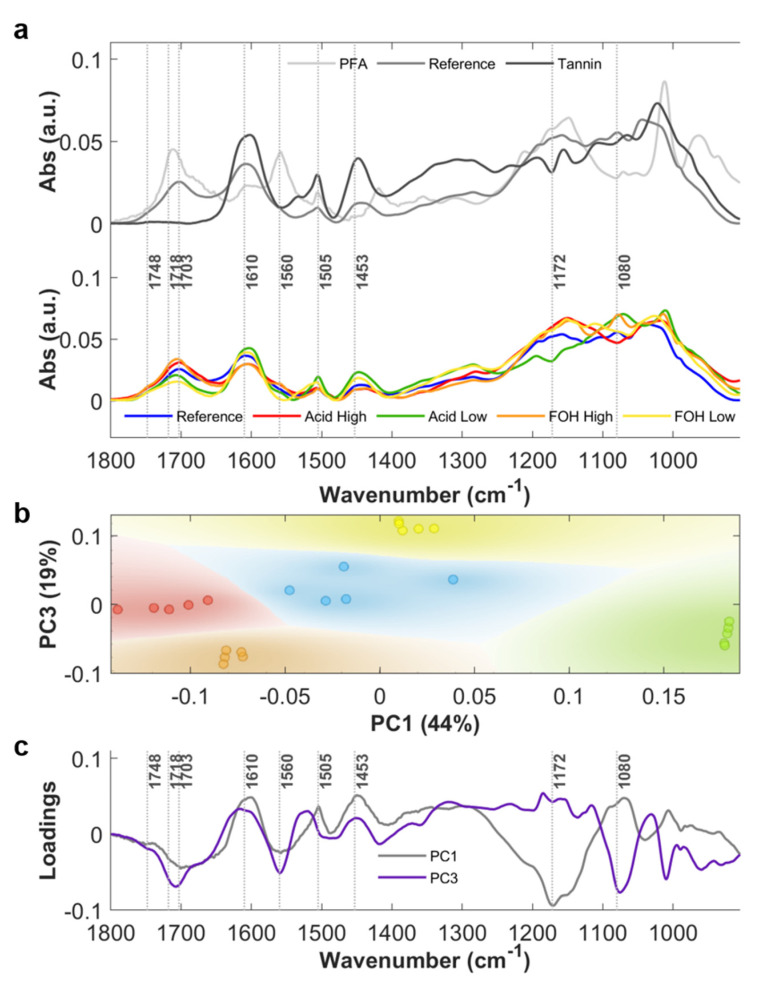
(**a**) Upper panel: Average absorbance spectra (*n* = 5) of tannin mimosa extract (Tannin), polyfurfuryl alcohol (PFA) and tannin-furanic reference foam (Reference); Bottom panel: Average absorbance spectra (*n* = 5) of tannin-furanic foam formulations: Reference (blue), Acid High (red), Acid Low (green), FOH High (orange) and FOH Low (yellow). (**b**) PC1-PC3 scatter plot. (**c**) PC1 and PC3 loadings, gray and purple lines respectively.

**Figure A1:**
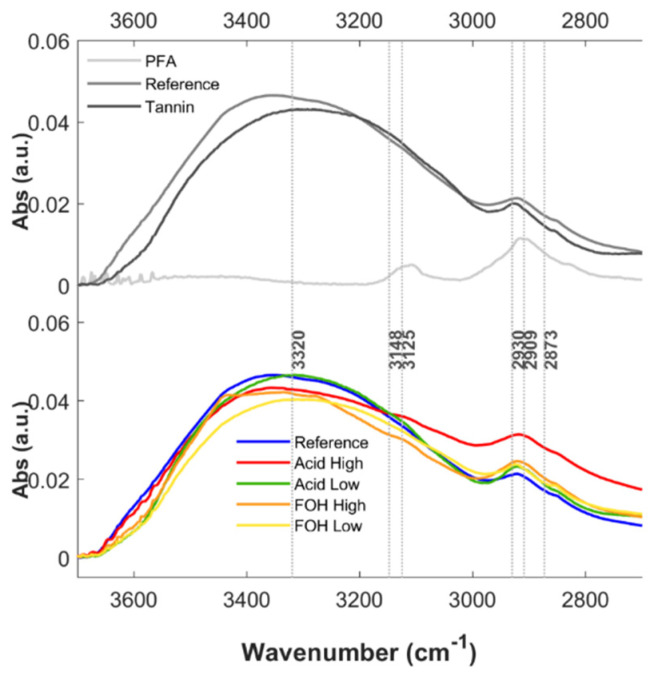
FTIR spectra of PFA, mimosa tannin extract and tannin-furanic foam (five formulations) in the 2600–3800 cm^−1^ spectral region. Upper panel: Average absorbance spectra of Reference foam (dark-grey), PFA (medium-grey) and mimosa tannin extract (light-grey). Bottom panel: Average absorbance spectra of Reference foam (blue), Acid high (red), Acid low (green), FOH high (orange) and FOH low (yellow) treatments in the 2800–3500 cm^−1^ region.

Average ATR-FTIR absorbance spectra of the crushed powders of the five considered formulations (Reference, Acid High, Acid Low, FOH High and FOH Low) are plotted in Figure 2a, bottom panel, in the 900–1800 cm^−1^ spectral region. In Figure S1a, a photo of the Reference rigid foam is shown, while Figure S1b shows the photos of the crushed powders. In Figure 2a, upper panel, the spectra of PFA and tannin mimosa extract are also shown for comparison with tannin-furanic foam Reference spectrum, in order to help the spectral interpretation. The same average spectra in the 2700–3800 cm^−1^ spectral range are plotted in Figure A1 of Appendix A. In Table 1, the attribution of the most relevant spectral bands of the tannin-furanic foam is given, based on the literature on the specific topic (See Table 1 references). More details on the spectral attribution and complexity on interpretation of tannin-furanic foam FTIR spectra are provided in Appendix A. For the purpose of the main manuscript, some IR signals of the foam in the 900–1800 cm^−1^ spectral region can be considered diagnostic of furanic moieties, such as the ones at ~1748, ~1718 and ~1703 cm^−1^, associated to carbonyl stretching, while the broader signal centered at ~1610 cm^−1^, associated to aromatic -C=C-, is mostly related to the phenolic nature of tannins. Therefore, under these assumptions, FTIR spectroscopy can provide evidences of the relative abundance of tannin and furanic blocks in the foam, but clear spectroscopy evidences of the extent of the copolymerization are not directly deducible. Indeed, methylene bridges are expected to link the copolymer blocks, but their quantification is actually prevented by the complex architecture of tannin rigid foam spectrum, being stretching and bending modes of methylene moieties also part of the tannin and PFA, both for the linear (I, II) and cyclic (III) forms.

Principal Component Analysis (PCA) of absorbance spectra in the 900–1800 cm^−1^ spectral region was exploited to highlight the most relevant differences in the 5-formulations dataset. The resulting PC1 vs PC3 scatter plot, shown in Figure 2b, reveals a clear separation between the different analyzed samples. The Reference foam stands in the center of the scatter plot whereas the treated samples are arranged around it. In particular, Acid High and Acid Low formulations are distributed along PC1 axis, while the formulations FOH High and FOH Low along PC3 axis. The corresponding loading vectors are shown in Figure 2c. In PC1 loading, the main contributions are at 1172 cm^−1^, related to C-O-C vibrations of Diels Alder arrangement of PFA, at 1560 cm^−1^, assigned to the C=C stretching of the 2,5-disubstituted furan ring, at the spectral frequencies associated to carbonyl moieties of PFA, and at 1453 and 1505 cm^−1^ due to the C=C aromatic stretching of different constituents of the foam, mainly of tannin origin. The trend reveals that for higher concentrations of the acid catalyst, the reaction path favors FOH polymerization with respect to the Reference formulation, while the aromaticity of polyphenols is reduced, possibly due to side reactions initiated by the strong acid catalyzer that induces the aromatic ring opening. When considering FOH diversified formulations, PC3 presents the most intense contributions at 1560 cm^−1^, at around 1703 cm^−1^ and 1714 cm^−1^, due to C=O of PFA, and at 1080 cm^−1^, associated to alicyclic secondary alcohol (pyranic ring). Therefore, at higher concentrations of FOH, the reaction conditions also favor the FOH polymerization, but without affecting the aromaticity of tannin.

Nevertheless, since clear spectroscopic evidences of the copolymerization are not directly achievable, it is impossible by the sole 1D chemical information to speculate on the polymer block’s alternation/graft in the copolymer structure. Therefore, 2D analysis was performed in order to understand if the spatial localization of furanic and tannin moieties at the micron-scale was comparable or not, being the spatial-heterogenicity an indirect consequence of prevalent segregation, while homogeneity of alternated copolymerization. 

### 2.2. 2D-FTIR Imaging of Tannin Foams

**Figure 3:**
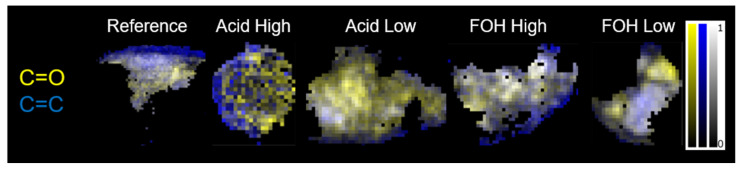
False color images at zero angle projection of the five formulations obtained by integrating vector normalized spectra for the ROI_1 (yellow) and ROI_2 (blue) upon 0–1 normalization of each channel. Black to white grayscale indicates the sum of the two channels.

FTIR images of the five formulations were collected on minutes foam pieces attached into nylon loops, as better specified in Section 4.3. FTIR hyperspectral images were generated integrating vector-normalized foam absorbance spectra, after removal of the loop spectral contribution (See Appendix B), on the most indicative spectral Regions of Interest (ROIs) of furanic and tannin polymer blocks, as retrieved by 1D analysis: the stretching band of carbonyl groups (1690–1730 cm^−1^), ROI_1, and the -C=C- chemical moieties (1580–1635 cm^−1^), ROI_2, respectively. The considered images are the first of the data set acquired for each sample for FTIR μ-CT purposes, and they will be named images at zero angle projection from here on. In Figure 3 are shown the 2D false colour pixelgrams, obtained by combing yellow (ROI_1) and blue (ROI_2) channels upon normalization of each between 0 and 1. Black to white regions highlight sample areas/hot-spots of colocalization of both moieties from lower to higher concentrations. Conversely, intense yellow regions and intense blue/violet regions indicate a predominance of furanic or tannin moieties respectively. 

Considering the Reference foam, distinct and extended tannin rich areas can be seen as blue and violet regions. Furanic and tannin moieties only partially colocalizes (black to gray pixels), letting to postulate the presence of variable-size furanic and tannin blocks, with a predominance of tannin ones. A mostly graft tannin-foam copolymer can be hypothesized also in the case of the Acid Low formulation: the large predominance of yellow areas allows to speculate that a dominant homopolymerization of FOH to PFA could have taken place. When considering the Acid High formulation, a tannin-rich area can be highlighted (blue peripheral region) with respect to a fragmented distribution of small tannin and furanic hot-spots in the inner part of the sample, and more extended furanic hot spots in bright yellow. The present results do not support our initial hypothesis of a prevalent grafted copolymerization resulting in a chemically homogenous foam in the case of Acid High formulation. 

For FOH High formulation, a clear co-localization of furanic and tannin hot-spots exists which appears in grayscale colour zones. Only sporadic segregated tannin and furanic richer areas can be highlighted as bluish and yellowish hot spots, differently from our initial hypothesis of more extended furanic blocks for this formulation. Conversely, for FOH Low formulations, a clear segregation between furanic and tannin blocks can be clearly distinguished (bluish and yellowish sample regions).

Nevertheless, the investigated samples are inhomogeneous in shape, both lateral size and thickness. It is therefore questionable if the observed hot spots derive solely from a different chemistry or they are also affected by polymer blocks segregation also along the *z*-axis.

### 2.3. 3D FTIR µ-CT

**Figure 4:**
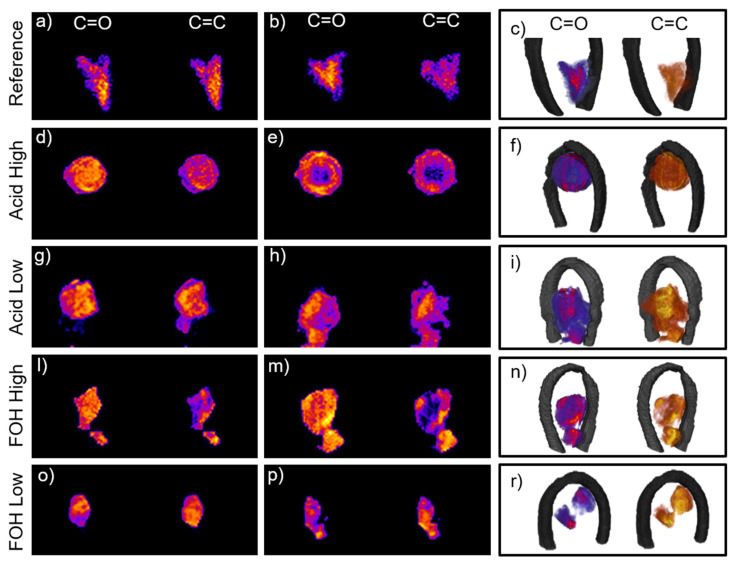
(**a**,**b**) Reference foam slices 96/150 and 68/150 from the full reconstruction in movie Reference_merged.mp4 in SI of the distribution of ROI_1 and ROI_2; (**c**) Corner-cut representation of the 3D reconstruction of the reference foam: the corner cut is generated by the intersection of two orthogonal planes in order to highlight the inner chemical distribution of the furanic areas (left in red-violet color scale) and tannin areas (right in yellow-orange color scale). From (**d**–**r**) same representation as (**a**–**c**) for Acid High (slices 50/150 and 65/150 from movie Acid_High_merged.mp4 in SI), Acid Low (slices 57/160 and 106/160 from movie Acid_Low_merged.mp4 in SI), FOH High (slices 73/100 and 55/100 from movie FOH_High_merged.mp4 in SI) and FOH low (slices 39/120 and 50/120 from movie FOH_Low_merged.mp4 in SI) formulations, respectively.

In order to further investigate the diverse formulations and clarify the doubts generated by the analysis of 2D images, FTIR µ-CT experiments were performed on the five foam formulations. In Supplementary Materials, the virtual sectioning of the five foams can be followed in the movies, for both ROI_1 and ROI_2. In Figure 4, selected virtual sections of the samples are showed, together with 3D renderings. For the Reference foam, the analysis of the movie allows to assert that furanic and tannin blocks mostly colocalizes at the diverse foam planes (see Figure 4a, slice 96/150 Movie Reference_merged in Supplementary Materials), while, only at specific sample planes, a clear segregation between regions richer in furanic moieties and exclusive tannin areas can be detected (see Figure 4b, slice 68/150 Movie Reference_merged in Supplementary Materials). The 3D reconstruction substantially highlights a more homogenous formulation for the reference foam with respect to 2D, while confirming the presence of distinct tannin blocks, which was the dominant information retrieved from planar images. For the Acid High formulation, the 3D-IR images reveal a situation very different form the one assumed by 2D ones. The movie Movie Acid_High_merged in in Supplementary Materials clearly highlights colocalization of the diagnostic moieties (see Figure 4d, slice 50/150 Movie Acid_High_merged in Supplementary Materials), supporting the initial hypothesis that a prevalent alternate copolymerization route took place in this case. In addition, the movie proves that the selected foam piece has a void region inside (see Figure 4e, slice 65/150 Movie Acid_High_merged in in Supplementary Materials). This evidence is not surprising due to the porous nature of the foam, but the presence of the large pore was not deducible from both optical and 2D FTIR images. Indeed, the pore presence can justify the overall lower intensity of the acquired spectra in the central region of the sample (data no shown) and led to conclude that the detected partial segregation of the furanic region in the 2D image was caused by an underestimation of the ROI_1 integral with respect to the more intense ROI_2 one as a consequence of the void. Acid_Low_merged movie in in Supplementary Materials clearly reveals the presence of segregated furanic and tannin regions, intercalated with regions of copresence, substantially confirming the 2D analysis, and better detailing it (See Figure 4g–h, slices 57/160 and 106/160 Movie Acid_Low_merged in in Supplementary Materials respectively). Conversely, the analysis of Movie FOH_High_merged of FOH-High formulation gives results that are not fully in-line with 2D hyperspectral image analysis. In this case, colocalized tannin and furanic hot spots can be seen, indicative of copolymerized areas (see Figure 4l, slice 73/100 sec Movie FOH_High_merged in Supplementary Materials), but also extended segregated furanic-regions that do not overlap with tannin ones (see Figure 4m, frame 55/100 Movie FOH_High_merged in Supplementary Materials). This evidence confirms our initial hypothesis on the effect of FOH increase concentration in the formulation, and supports the explanation of 1D analysis. Also, for the FOH-Low formulation, copolymerizing-regions coexists with segregated hot-spots of both furanic and tannin nature (see Figure 4o,p, slices 39/120 and 50/120 Movie FOH_Low_merged movie in Supplementary Materials). Similar considerations can be drawn also analyzing the corner-cut 3D images presented in Figure 4c,f,i,n,r. In this case a section of the reconstructed volume has been virtually removed along two orthogonal planes intersecting at the center of the sample. This allows to observe at one glance the variation of the C=O and C=C distribution from outside to inside of each synthesis product.

Overall, the progression from 1D to 3D analysis confirms the hypothesis on the effects of the diverse formulation on the final chemical composition and spatial organization of the tannin foam, both characteristics that could not be obtained by 2D analysis alone. Indeed, with respect to 2D analysis, the 3D reconstructions provide also the first evidences on the fact that average foam formulation is not the same at each foam location, and that the variability seems independent to the foam morphology since tannin and furanic regions segregate or coexist in the foam cells at unspecific locations. This information could be clearly deduced only from 3D µCT, since the projection of a 3D object on a 2D plane could lead to partial conclusions. 

**Figure 5:**
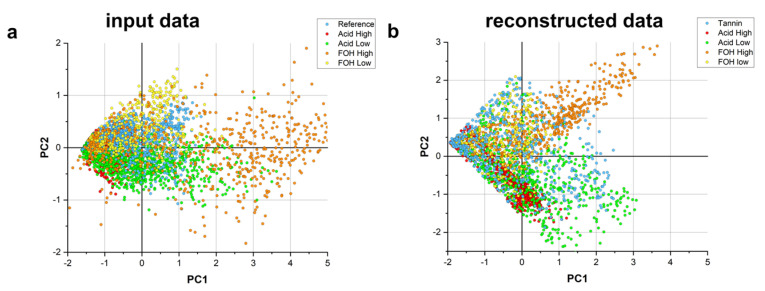
Comparison of the PCA obtained using the input data (panel **a**) and the reconstructed data projected and summed along the transversal plane at angle zero (panel **b**) PC1 and PC2 are the same for the plots, PC1 0.7 C=C, 0.7 C=O and PC2 0.7 C=C, −0.7 C=O.

Nevertheless, one might doubt on the consistency of the results inferred from 3D chemical images, considered the heavy data processing applied for their reconstruction. Therefore, to confirm the limited effect of the data treatment on the chemical information contained in the original data, the spectral band integrals of ROIs along the projection axis were computed at zero projection angle (2D sum images in Section 4.3) as described in Section 4.3, and used as input for PCA analysis. The results were compared with the ones obtained by the integration of the same ROIs of the raw 2D images at zero projection angle, after nylon loop removal. In Figure 5, the PC1-PC2 scatter plots for the two datasets are reported. For both datasets the PC1 and PC2 are a composition of the C=C and C=O integral values, with the same coefficients, with PC1 directly proportional to C=C and C=O and PC2 directly proportional to the values of C=C and inversely proportional to the C=O. The most evident differences rely on the sharp boundaries of 2D sum images, due to the image threshold for 3D data processing workflow (see Appendix B for more details), and on the slightly different orientation of FOH High formulation with respect to PC2. Despite these differences, the comparison of the scatter plot highlights that the information content of reconstructed 2D images qualitatively matches the chemical heterogeneity retrievable for 2D raw images and indirectly confirm that the adopted image processing, does not alter the qualitative chemical information.

## 3. Conclusions

Here, we report on a multidimensional FTIR 1D-2D-3D analysis of tannin-furanic rigid foams, a green material for which, many aspects of the foam production remain unclear. First of all, its complex polymerization chemistry and how the foam composition might be affected by the possible formulations and synthesis conditions, both on average and locally. We present a systematic study on diverse foam formulations, covering aspects related to the diverse foam chemistry in a spatially resolved manner. Thanks to the FTIR-µCT, a volumetric representation of the distribution of the chemical species inside the foams was achieved, letting us first present a 3D chemical representation of tannin-furanic foams and highlight their spatial heterogeneity at the microscale. By focusing on 3D data treatment reliability, to the best of our knowledge, this work presents for the first-time evidences on the trustworthiness of FTIR-µCT results, for a complex material for which there are no obvious correspondences between optical and chemical images, as clearly proven for example by the Acid High formulation foam.

Overall, the present paper goes beyond a simple proof of principle of FTIR-µCT and proves its value for chemically complex and heterogeneous materials. Indeed, rigid tannin-furanic foams are just an example of a class of materials that can take advantage from FTIR-µCT: extremely light and porous materials that can be investigated in relatively large and representative pieces by this technique. Most of them are technologically relevant in many fields, and can become a key-topic for FTIR-µCT applications. It is indeed sure that with further improvements on the detector side, with brighter sources, advanced opto-mechanical setups and more automated data processing, FTIR-µCT can grow into an important analytical tool to routinely use for investigating complex samples and provide new insights about their 3D chemical distribution at the microscale.

## 4. Materials and Methods

### 4.1. Sample Preparation

Five tannin-furanic foams were produced by mixing the powdered tannin extracted from Acacia mimosa ((Tanac SA, Montenegro, RS, Brazil) with water and furfuryl alcohol (Transfurans Chemicals, Geel, Belgium). Sulfuric acid (32%, Merck, Darmastadt, Germany) was added to the reagents’ mixture until it was homogeneous and was then placed in the oven for 30 min at 90 °C. Afterwards, the mixture was kept at 20 °C and 65% relative humidity to fully react, until achieving the classical appearance: a dark-brown rigid foam. The complete recipe for reference tannin foam is reported elsewhere [31], whereas the four other foams were synthesized by changing the FOH concentration or the acid concentration on each one with respect to the reference formulation, as reported in Table 2.

### 4.2. ATR-FTIR Measurements

The foams were crushed into powder, and then measured using the monolithic diamond ATR accessory Platinum (Bruker Optics) of the Vertex 70v interferometer (Bruker Optics) in vacuum, equipped with the FIR-MIR DTGS detector and beamsplitter. For each formulation, *n* = 5 different samples were measured in the 50–6000 cm^−1^ range, averaging 256 scans at 2 cm^−1^ spectral resolution. The recorded spectra were cut in the 400–4000 cm^−1^ range, baseline corrected (concave rubberband method: number of iterations = 5, number of baseline points = 64), vector normalized and offset corrected in the whole range. FTIR-ATR spectra analysis was performed using Quasar [32]. Second derivative was calculated using the Savitzky-Golay filter (3rd order polynomial, 21 smoothing points). PCA analysis of the vector normalized absorbance spectra was performed in the 900–1800 cm^−1^.

### 4.3. FTIR-µCT Data Acquisition

FTIR-µCT measurements were done both at the Chemical and Life Science branch of SISSI beamline [33] and the INFN-LNF DAθNE-Light Facility in Frascati (Rome) [34]. For the purposes of the measurements, the Vis-IR 3000 Hyperion microscope, equipped with 64 × 64 pixels FPA detector and coupled with VERTEX70v interferometer, was used (Bruker GmbH). Measurements were done in transmission mode, 15× Cassegrain objective/condenser (NA = 0.4). The FPA detector pixel resolution in this configuration is about 2.7 × 2.7 μm^2^. 

Before the experiment, a single small piece of the sample was attached to a nylon micro-loop (50 µm aperture size and 20 µm nylon rod diameter): the fragment was fished from a NaCl (0.9%) solution and, by exploiting the evaporation of the solvent and the crystallization of the NaCl salt, the specimen was anchored onto the loop. The tomographic setup is shown in Figure S2 and described in the Figure caption. In Supplementary Materials, Movie clip 1 shows the setup in operation. More technical details on the FTIR-µCT system here used can be found elsewhere [10]. Movie clip 2 in Supplementary Materials zooms the nylon loop through the visible camera and the FPA.

Each hyperspectral projection was acquired averaging 128 scans at 16 cm^−1^ spectral resolution in the 900–4000 cm^−1^ spectral range, in order to limit the acquisition time to 80 s per image. For the same reason, only the sample interferogram was recorded and the Fourier Transform (FT) and the rationing with an air background in order to obtain the absorbance spectrum was performed afterwards. Spectral acquisition and FT (zero filling factor: 2; apodization function: Blackman-Harris 3-Term; phase correction mode: Mertz; non-linearity correction: 1) were done by using the Bruker proprietary software OPUS 7.5.

Briefly, once recorded a single FTIR-FPA hyperspectral image, the sample was rotated by 1°, until having acquired the whole dataset: 360 projections were collected. Each sample experiment took about 8 hours, although, being the procedure completely automated and motion and acquisition software fully synchronized (See Appendix B for more details), it can be left unattended.

The first FPA image of each dataset, namely zero angle projection, was individually used for the purposes of 2D-image analysis.

### 4.4. FTIR-µCT Data Processing

**Figure A2:**
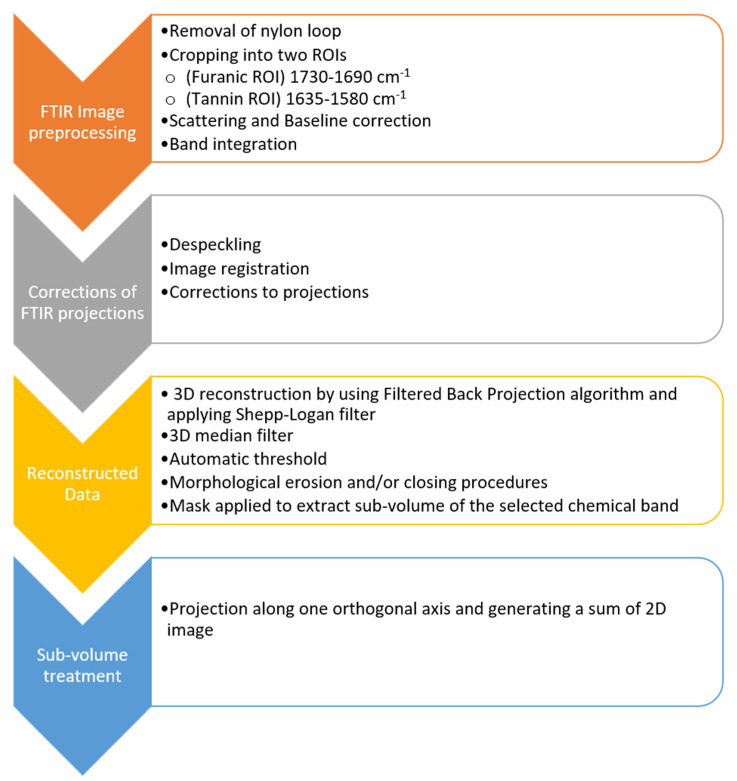
3D FTIR μ-CT data processing workflow data processing workflow.

**Figure A3:**
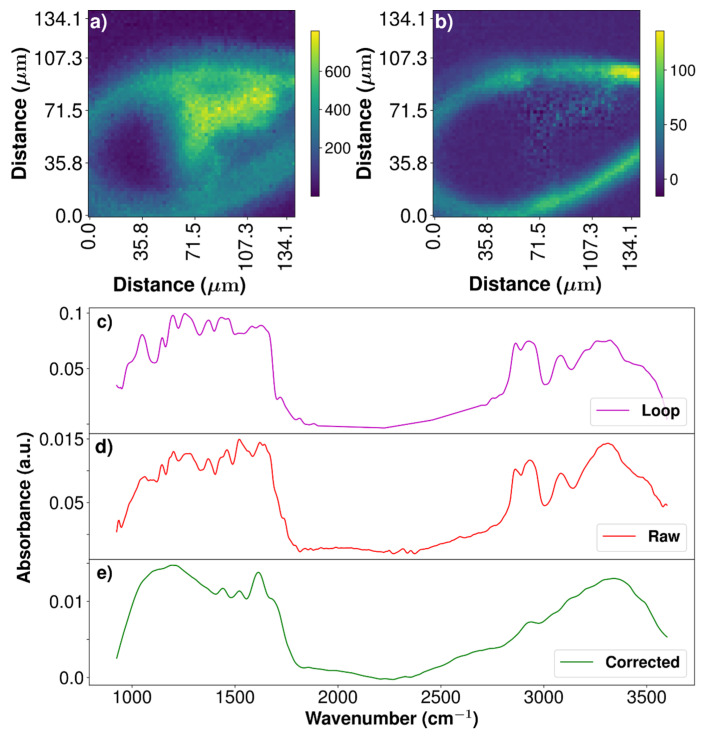
(**a**) Full range (900–3600 cm^−1^) integration of a reference map. (**b**) Loop identification as extracted using the developed software: from the correction output, the loop contribution coefficients were taken and used for 3D rendering of the sample support. (**c**) Average spectrum of the nylon loop (**d**) Raw spectrum (**e**) Corrected spectrum as obtained using the developed software.

The data processing workflow is summarized in Appendix B and sketched in Figure A2 and Figure A3. Briefly, FTIR image pre-processing and post-processing were fully automated in order to output 2D corrected images in the Regions of Interest (ROIs) for the present paper: ROI_1: 1690–1730 cm^−1^ (ν(C=O) - stretching mode of carbonyl groups); ROI_2: 1580–1635 cm^−1^ (ν(C=C) - stretching of the aromatic rings). All the procedures were executed by an in-house code written in Python 3.5 using the routine matplotlib [35], numpy [36], and scipy [37] packages. CT reconstruction was then performed according to X-ray micro-CT standards, as detailed in Figure A2 caption.

Finally, for each rendering, the sub-volume of each ROI was projected along the axis corresponding to the optical path of the zero-angle projection, to generate a 2D image (or equivalently the integral along the projection axis). These images were then compared with the FTIR-µCT pre-processed images at 0 degrees through PCA analysis. 

## Figures and Tables

**Table 1:** Attribution of the most relevant spectral bands of the tannin-furanic foam. List of abbreviation: str. = stretching; asym. = asymmetric; sym. = symmetric; def. = deformation; Ar = Aromatic; scis. = scissoring; vib. = vibration.

Band [cm^−1^]	Vibrational mode	Assignment
3360	-OH str.	Polyphenolic matrix
3100	=C-H str.	Aromatic C-H [22,23,24]
~2930, ~2850	CH_2_ asym., sym str.	Methylene group [22]
1748, 1718, 1703	C=O str.	α,β-unsaturated γ-lactone [19] and ketones
1626, 1620, 1616	-C=C- Ar. str.	Catechin [25], Catechol structure (ring B) [22], Epicatechin [25]
1610	-C_4_-C_8_- str.	Linking bond between flavanol monomers [26]
	-C=C- Ar. str.	Resorcinol structure (ring A) [22]
1595, 1560, 1505	-C=C- Ar. str.	Catechol, resorcinol and phenols [22], 2,5-disubstituted furan ring in PFA [23], 1,2,4-trisubstituted aromatic ring, cathecol [23]
1453, 1435	-C=C- Ar. str.	Phenols
	-C-H def. [23]	Aliphatic chains
	-CH_2_ scis. [26]	Pyranic ring
1360, 1325	C-OH def.	Phenolic compounds
1285	-C_(Aryl)_-O_(Pyranic)_- asym. str.	Pyranic ring [27]
1230, 1195,1157	C-OH str.	Phenolic compounds
1175	C-O-C vibrations	Diels Alder arrangement PFA [19]
1105	-C_(Alkyl)_-O_(Pyranic)_- asym. str.	Pyranic ring
1080	C-OH str.	Alicyclic secondary alcohol (pyranic ring)
1048	C-C str.	Skeletal vibrations [22]
	C-O asym. str.	Polyphenols [28,29]
1014, 960, 880	C-H vib.	Furanic ring [19,30]
960, 845	C-H out of plane ben.	Aromatic rings [22,27]

**Table 2:** Formulations of tannin-furanic foams.

	Tannin [g]	H_2_0 [g]	FOH [g]	H_2_SO_4_ [g]	Temp [°C]
Reference	5	0.93	3.15	2.22	90
Acid High	5	0.93	3.15	4.44	90
Acid Low	5	0.93	3.15	0.22	90
FOH High	5	0.93	6.30	2.22	90
FOH Low	5	4.00	0.32	2.22	90

## Data Availability

Raw FTIR data were generated at Elettra Sicnrotrone Trieste in Trieste (Italy) and INFN-LNF DAθNE-Light Facility in Frascati (Rome). Derived data supporting the findings of this study are available from the corresponding author G.B. on request.

## Supplementary Materials

Supplementary materials can be found at https://www.mdpi.com/article/10.3390/ijms222312869/s1.

## Appendix A

Appendix A aims to detail the spectral assignments of Table 1, referred to tannin-furanic foam spectra, also considering the spectra of mimosa tannin extract and PFA shown in Figure 2a, upper panel. Furthermore, it considers the 2700–3600 cm^−1^ spectral region, as shown in Figure A1, upper panel. In Figure 2a, bottom panel, and A1, bottom panel, the spectra of the five formulations are also shown for the same spectral ranges. The interpretation is given on the base of literature papers, published by the authors and other research groups. It has to be highlighted that, due to the complexity of the polymerization pathways, roughly sketched in Figure 1, and the variability introduced by the polymerization conditions, a unified vision on the tannin-furanic foam chemistry has not been achieved yet.

Considering the high-frequency spectral region (Figure A1), a broad peak at 3360 cm^−1^ is observed on the Reference foam, and it originates from the stretching vibrations of the O-H bonds present in the polyphenolic matrix, as can be clearly appreciated from the comparison with PFA and mimosa tannin extract spectra. The co-presence of the several not equivalent hydroxyl groups on the aromatic rings as well as on the pyranic one justifies the broadening of the signal, a phenomenon that is also influenced by hydrogen bonding network, specifically in the solid phase. The region is therefore potentially diagnostic of polyphenol blocks in the foam, but since this spectral region was saturated in many images, it was considered not fully reliable. Comparing the Reference foam spectrum with the ones of the four tested formulations, it can be deduced that the observed variations depending on the different synthetic products are not highly informative on the differences between the diverse average formulations. Also, for this reason, this spectral range has not been considered for further analysis.

Due to the broadening effect of the aforementioned -OH stretching peak, the signal at around 3100 cm^−1^, assigned to the stretching of aromatic C-H, is usually not clearly detectable in both tannin extract and foam spectra, or it appears as a small shoulder [22,23,38]. The peaks due to the asymmetric stretching of aliphatic C-H are instead visible and separated from the previous signal in the 2800–3000 spectral region. Nevertheless, the methylene stretching bands are very broad due to the multiple nature of methylene moieties in both PFA, polyphenols and foams [22]. Therefore, despite these bands could be indicative, in position and intensity, of the polymer block linkage, to retrieve any useful information in this respect is nearly impossible from the sole FTIR analysis.

Focusing on the 1680–1750 cm^−1^ spectral region, second derivative analysis of the Reference tannin foam spectra (data not shown) highlights that the signal is composed of three peaks (~1748 cm^−1^, 1718 cm^−1^ and 1703 cm^−1^), indicating the presence of three different carbonyl moieties. The peak at 1748·cm^-1^ is also present in the PFA and associated to the C=O stretching of α,β-unsaturated γ-lactone as terminal monomer of the polymer chain [19]. The peaks at 1718 cm^−1^ and 1703 cm^−1^ are due to the carbonyl groups associated to the presence of ketone moieties of different PFA homopolymers, such as the linear and open-ring forms as showed in Figure 1. Only small variations between the PFA and Reference tannin-furanic foam spectra can be noticed in this spectral region, and it stands in the relative intensities of the aforementioned peaks: in PFA the dominant peak is at 1718 cm^−1^, while for the tannin foam the band component higher in intensity lies at 1703 cm^−1^. Indeed, the presence of the peak is important to distinguish hydrolysable tannins from the condensed ones as reported in literature [39,40]. A straightforward explanation of the trend is not trivial, but indeed we can safely assume that the spectral range is indicative of polymer furanic-blocks. This is also confirmed by the fact that increasing the concentration of the furfuryl alcohol in the reaction mixture the overall peak shifts from the 1703 cm^−1^ to the 1718 cm^−1^ position.

In the 1435–1620·cm^-1^ region, there are several peaks, mostly associated with the -C=C- stretching vibrations of the aromatic rings. In this respect, the main actors are the furanic ring of the furfuryl alcohol as well as the catechol, resorcinol and pyrogallol polyphenolic structures of the tannin extract. Depending on the number and the position of hydroxyl groups, the peaks change the vibrational frequencies. In particular, resorcinol structure (ring A) generates a peak at 1610 cm^−1^ while the catechol moiety (ring B) vibrates at 1620 cm^−1^ [22]. In addition, the diastereoisomers composing the tannin blend are responsible for a gentle broadening of the peaks: in particular this is true for the peak at around 1620 cm^−1^. A computational study reports that resorcinol rings in catechin structures vibrate at 1626 cm^−1^ while in epicatechin ones the signal is present at lower wavenumbers (1616 cm^−1^) [25]. In real cases, it is particularly difficult to observe the presence of two peaks rather than a single one, raising from the contribution of both the diastereoisomers. The peak at around 1595 cm^−1^ might be not visible or present as a shoulder of the peak at 1617 cm^−1^. It characterizes cathecol, resorcinol and phenol-like compounds and it is usually not affected by the poly-substitution of the aromatic ring in both position and intensity [22]. The peak at 1560 cm^−1^ is associate to the polymerization product of PFA. The peak at 1505 cm^−1^ is assigned to the polyphenol carbon-carbon double bonds belonging to the 1,2,4-trisubstituted aromatic ring (ring B: cathecol) [23]. Overall, the present analysis highlights that the spectral region 1580–1635 cm^−1^ can be considered mostly diagnostic polyphenol blocks. Two more signals were identified at 1453 cm^−1^ and 1435 cm^−1^ and assigned to several vibration modes as C=C aromatic stretching, aliphatic C-H deformation [23] and CH2 scissoring of the pyranic ring [41].

In the 900–1360 cm^−1^ region, the predominant vibrations involve the C-O and C-C stretching modes as well as the C-OH phenolic stretching and bending deformations and C-H aromatic bendings. Due to the chemical complexity of the polymer the assignment of each peak to a specific vibrational group is not straightforward. Signals at 1360 and 1325 cm^−1^ are associated to both the C-OH bending deformation [23,38] and the aromatic C-H bending [19] while at lower frequencies it is possible to assign the 1230 cm^−1^, 1195· cm^-1^ and 1157 cm^−1^ peaks to the C-OH stretching in phenol compounds. In the middle of those, the peak at 1285 cm^−1^ in literature is commonly assigned to the asymmetric stretching between aryl carbon and oxygen of the pyranic ring [27]. On the other side, the ether-like stretching between alkyl carbon and oxygen of the same pyranic ring is located at the lower wavenumbers, most likely at 1157 cm^−1^ or at 1105 cm^−1^. The peak at 1175 cm^−1^ is associated to the C-O-C vibrations in Diels-Alder arrangement of PFA chains as reported elsewhere [26]. Alicyclic secondary alcohol of the pyranic ring should be in the 1030–1085 cm^−1^ range and therefore, the 1080 cm^−1^ or 1048 cm^−1^ peaks are both reasonably valid for the assignment. We propose that the peak at higher wavenumbers is the C-OH stretching because in the acid high formulation decreases in intensity while the peak at 1105 cm^−1^ increases: this might be due to the etherification of the secondary alcohol in acidic conditions and at suitable temperatures (<130 °C). At the same time the peak at 1048 cm^−1^ is reported to be due to the C-C skeletal stretching modes, even though some authors assign the peak close to this wavenumber to the C-O asymmetric stretching of phenolic moieties [28,42]. Peaks at 1014 cm^−1^, 960 cm^−1^ and 880 cm^−1^ are mostly generated by the PFA vibrations as reported by Tondi et al. [19,30]. At the same time the peaks at 988 cm^−1^ and 845 cm^−1^ are identified as C-H aromatic out of plane bending and specific to the tannin moiety [29].

## Appendix B

Appendix B aims to detail the 3D FTIR μ-CT data analysis workflow, according with the processing steps sketched in Figure A2. **FTIR image pre-processing**: data analysis of the FTIR imaging datasets (projections) was found to be a challenging issue due to considerable scattering contributions and in some cases a high level of contamination by the nylon loop used for sample anchoring. The entire dataset was first subject to spectral correction to remove the contribution of the nylon loop from every FTIR spectrum, as previously optimized [10]. The results of spectral correction and loop contribution removal are summarized in Figure A3. Next, the corrected spectra were cropped to selected spectral Regions of Interest (ROIs) indicative of furanic-blocks, 1690–1730 cm^−1^ (ν(C=O)-stretching mode of carbonyl group), and polyphenol- blocks, 1580–1635 cm^−1^ (ν(C=C) aromatic stretching). The truncated data were then baseline-corrected with the linear baseline, and finally subjected to the band integration by the basic trapezoidal method. The same data analysis workflow was applied for each single FTIR-FPA projection image. All the procedures were assisted by the in-house code written in Python 3.5 with the routine matplotlib [35], numpy [36], and scipy [37] packages. **Corrections of FTIR projections**: Before the CT reconstruction, the FTIR projections were filtered by using a de-speckling procedure described by Brombal et al. [43] to remove pixels for which the integral value was either too low or too large when compared to the neighboring pixels. These speckles may result in streaking artifacts in the reconstructed images. In brief, a filter window of 5 × 5 pixels square is selected around each pixel of the 2D image, and the average and standard deviation of the window are computed. If the selected pixel differs from the average window value of more than 5 standard deviation window, then it is replaced by the window average value. Additionally, an automatic image registration is performed to compensate for the small motion artifacts during acquisition using a rigid body correction. Sample movements were computed starting from the analysis of the nylon loop since it has a known, rigid and consistent regular shape and it is morphologically homogeneous. The image correction transformations found from the loop were then applied to correct movements on the other tomographic data. Corrections to the projections were needed in order to compensate for small drifts happening during the rotation of the sample. The corrections consisted of finding x and y translations obtained from an automatic registration based on the previous projection. This procedure was generally applied automatically although fine tuning had to be performed manually in some circumstances. **Reconstructed data**: Each set of corrected projections was then reconstructed by using a Filtered Back Projections algorithm and applying a Shepp-Logan filter [44]. The output has then arbitrary values proportional to the IR signal in the selected ROIs. The sample center of rotation was found using the loop as the reference image. The reconstructed data were then post-processed using Avizo© FEI software. For each dataset, a 3D median filter was applied to the digital volume to compensate for image noise. Then, an automatic thresholding procedure [42] was used to segment voxels belonging to the sample. Additional morphological erosion and/or closing procedures were applied case by case to compensate for over- or under- segmented volumes. In addition, voxels intersecting those of the loop were removed. The binary image obtained was used as a mask to extract the sub-volume corresponding to the selected chemical band; the remaining voxels were then set to zero. **Sub volume treatment**: The sub-volume for each band is projected along one of the three orthogonal axes to generate the sum 2D image (or equivalently the integral along the projection axis). This image is then compared with the FTIR-µCT pre-processed image at 0 degrees.
