# Supplementary material for: Study of the Spatio-Chemical Heterogeneity of Tannin-Furanic Foams: From 1D FTIR Spectroscopy to 3D FTIR Micro-Computed Tomography
Source: Int J Mol Sci. 2021 Nov 28;22(23):12869. doi: 10.3390/ijms222312869 (PMC8658003; doi:10.3390/ijms222312869)
Supplement: Supplementary file 1 [file ijms-22-12869-s001.zip › Supporting Information_MDPI_Revised.pdf]

# Supplementary Materials

# Study of the spatio-chemical heterogeneity of tannin-furanic foams: from 1D FTIR spectroscopy to 3D FTIR micro-computed tomography.

Nicola Cefarin[1,2‡], Diana E. Bedolla[1,3‡], Artur Surowka[1,4‡], Sandro Donato[5,6‡], Thomas Sepperer[7,8], Gianluca Tondi[7,9], Diego Dreossi[1], Nicola Sodini[1], Giovanni Birarda[1,*] and Lisa Vaccari[1]

1 Elettra-Sincrotrone Trieste, S.S. 14 Km 163.5, 34149 Basovizza, Trieste, Italy
2 IOM-CNR, Trieste, S.S. 14 Km 163.5, 34149 Basovizza, Trieste, Italy
3 Area Science Park, Padriciano 99, 34149 Trieste, Italy
4 AGH University of Science and Technology, al. Mickiewicza 30, 30-059, Kraków, Poland
5 University of Calabria, Department of Physics, Via P.Bucci 31C, 87036, Rende (CS), Italy
6 Istituto Nazionale di Fisica Nucleare, Division of Frascati, Via Fermi, 54, 00044 Frascati, Rome, Italy
7 Salzburg University of Applied Sciences, Forest products technology & Timber constructions dept., Marktstrasse 136a, 5431, Kuchl, Austria
8 Salzburg Center for Smart Materials, Jakob-Haringerstrasse 2a, Salzburg 5020, Austria
9 University of Padua, Department of Land, Environment, Agriculture & Forestry, Viale dell'Università 16, 35020, Legnaro (PD), Italy

*Correspondence: giovanni.birarda@elettra.eu;

**Figure S1.** Tannin-furanic rigid foam.

**Figure S2.** FTIR-μCT setup.

**Movie Clip 1.mp4.** FTIR-μCT setup in operation.

**Movie Clip 2.mp4.** Nylon loop through the visible and IR camera (FPA).

**Virtual sectioning of the tomographic samples: Video Reference_merged.mp4: tannin-furanic foam; Video Acid_High_merged.mpa4: Acid-High formulation; Video Acid_Low_merged.mp4: Acid-Low formulation; Video FOH_High_merged: FOH-High formulation; Video FOH_Low-merged: FOH-Low formulation.**

**Figure S1. Tannin-furanic rigid foam.**

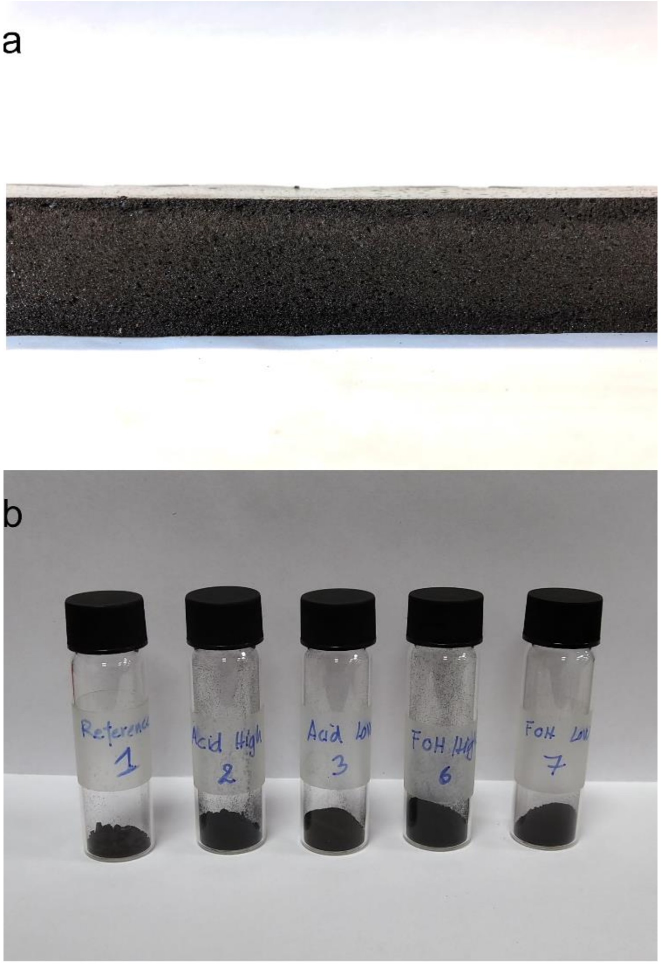


**Figure S1** a) Tannin-furanic rigid foam, Reference formulation b) Crushed powders of the five formulations as used for FTIR-ATR, FTIR imaging and FTIR-μCT: 1: Reference, 2: Acid High, 3: Acid Low, 6: FOH High, 7: FOH Low.

**Figure S2. FTIR μ-CT setup**

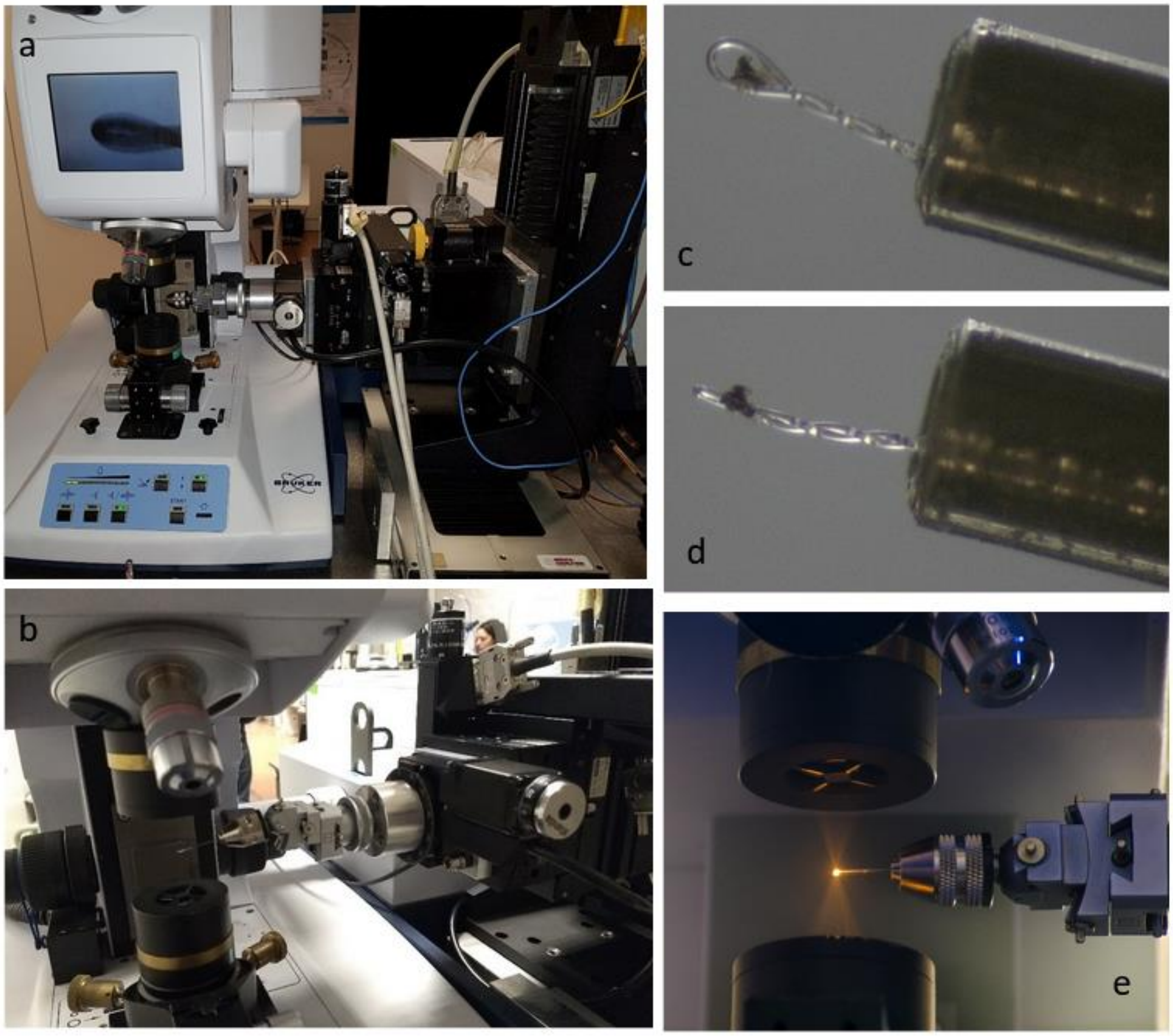


**Figure S2** (a,b) The tomography setup has been assembled using the following components: three independent Mdrive (M), Corvus (C) and Standa (S) motors, covering 6 degrees of freedom, enabled sample rotation (S), translation up/down (M1), translation inside-outside the Field of View - FOV (manual), pitch (C), roll (C) and drag (C) movements in the FTIR-μCT setup. Motor control and data acquisition procedures were integrated and automatized by a dedicated software written in LabVIEW 2017 utilizing USB (S) and TCP/IP (M and C) connection protocols. The software enabled full synchronization of sample rotation with the ongoing data acquisition with the OPUS 7.5 software.
